# Current and future directions for research on hallucinations and delusions

**DOI:** 10.1038/s41598-024-57472-6

**Published:** 2024-04-10

**Authors:** Reshanne R. Reeder

**Affiliations:** Department of Psychology, Institute of Population Health, University of Liverpool, Liverpool, UK

**Keywords:** Human behaviour, Preclinical research

## Abstract

Hallucinations and delusions can be symptoms of psychiatric illness, but more often—though less commonly known—are actually part of a healthy range of experiences found throughout the general population. The studies in this Special Collection paint a picture of the wide range of hallucinatory and delusional experiences across diverse populations, as well as comparative perspectives between clinical and non-clinical samples. In this editorial, I make three related points that are exemplified in the articles published here. First, that hallucinations and delusions are part of a normal distribution of human diversity; their mere presence does not indicate psychosis or psychiatric illness. Second, that the ubiquity of hallucinatory and delusional experiences across clinical and non-clinical populations suggests common cognitive and neural mechanisms. Finally, despite these commonalities, it is important to understand the difference between psychiatric symptoms and healthy experience. In summary, I conclude that it is important to investigate both common mechanisms and distinguishing factors to comprehensively elucidate these oft-misunderstood experiences. This Special Collection provides a showcase of the cutting-edge research that encompasses these objectives.

Hallucinations and delusions are two cornerstones of psychosis, a collection of symptoms that can occur across a range of psychiatric disorders and stems from a reduced ability to disentangle reality from fantasy^1,2^. Although it may seem that a Special Collection called *Hallucinations and Delusions* would mainly report studies of psychosis, these experiences are surprisingly common throughout the general population. So although psychosis is defined by hallucinatory and delusional experiences, the presence of hallucinations and delusions are not indicative of a psychotic disorder. Together, the studies in this Special Collection paint a picture of the wide range of hallucinatory and delusional experiences across diverse populations, as well as comparative perspectives between clinical and non-clinical samples. In this editorial, I would like to make three related points that are exemplified in the articles published here:

1. Hallucinations and delusions are part of a normal distribution of human diversity; their mere presence does not indicate psychosis or psychiatric illness.
2. The ubiquity of hallucinatory and delusional experiences across clinical and non-clinical populations suggests common cognitive and neural mechanisms.
3. Despite the aforementioned commonalities, it is important to understand the difference between psychiatric symptoms and healthy experience.

## A normal distribution of human diversity

**Hallucinations and delusions are part of a normal distribution of human diversity; their mere presence does not indicate psychosis or psychiatric illness. **Experimentally-induced hallucinations are safe, controlled, and have no negative consequences^3^; confabulating memories sometimes is perfectly normal^4^; and despite the insistence of some individuals, simply having conspiracy beliefs is not a sign of pathology^5^.

The study by Shenyan et al.^3^ demonstrates that hallucinations can be reliably induced in non-clinical samples using experimental techniques called Ganzfeld^6^ and Ganzflicker^7,8^. Prolonged, unstructured (Ganzfeld) or repetitive (Ganzflicker) stimulation to the visual system can elicit both simple and complex hallucinatory experiences, such as geometric patterns, illusory colors, or even meaningful real-world objects and scenes. The authors, for the first time, quantified the onset and frequency of Ganzfeld- and Ganzflicker-induced hallucinations, and included participant drawings to reveal diverse, individualized visual experiences.

In another study highlighting psychosis characteristics in the general population, Stephan-Otto et al.^4^ found that hallucination proneness is associated with false memories of novel words during a word recall task. The researchers asked participants to complete a series of questionnaires, memorize lists of high- and low-frequency words, then tested their recall while collecting fMRI data. Behaviorally, hallucination scores correlated with response bias for both high- and low-frequency words. Interestingly, neuroimaging data revealed a significant association between specifically verbal hallucination proneness and activation of brain areas related to language during false recognition of novel words. This points to individual differences in susceptibility to confabulated inner speech in the general population.

Finally, conspiracy beliefs are generally related to psychopathological characteristics (e.g., paranoia, anxiety) and erratic behavior such as volatility in decision-making^9^; however, in a paper published in this Collection, Suthaharan and Corlett^5^ found that these negative effects are reduced in individuals who have a strong social network around their beliefs. This interestingly suggests that social and environmental factors contribute to the (positive or negative) impact of conspiracy beliefs on mental health and behavior. In contrast to the stereotype of a ‘crazy conspiracy theorist’, the authors conclude that conspiracy beliefs are not inherently pathological.

## Common cognitive and neural mechanisms

**The ubiquity of hallucinations and delusions across clinical and non-clinical populations suggests common cognitive and neural mechanisms.** We are developing techniques to experimentally induce hallucinations, which could potentially elucidate how they can develop into psychotic experiences^3,7,8^. In the study by Shenyan et al.^3^, the authors purport that a (cortically hierarchical) low-level hallucinatory mechanism may be responsible for simple hallucinations, whereas top-down processes (such as mental imagery or beliefs) may contribute to complex hallucinations. These same mechanisms are proposed to be involved in different kinds of hallucinatory experiences in clinical populations, as well^10^. Further, Stephan-Otto and colleagues^4^ propose common mechanisms for reality monitoring of inner speech in both clinical and non-clinical populations. These techniques could therefore be used to better understand the cognitive and neural mechanisms that contribute to hallucinatory experience across diverse populations.

Comparative studies in this Collection highlight similarities in the content and organization of psychotic symptoms across different populations. For example, Fleming and colleagues^11^ modeled a data-driven profile of symptoms experienced in first-episode psychosis (FEP) and persistent psychotic illness. Individuals with FEP are those that have only recently begun to experience symptoms of psychosis, which may or may not progress into persistent psychotic illness, specifically schizophrenia or schizoaffective disorder. Interestingly, the multidimensional profile of symptoms is quite similar between the two groups: auditory hallucinations tended to cluster with delusions that the patient was under another agent’s control; religious and grandiose delusions both clustered with thought disorder symptoms; and other reality and perceptual distortions formed a separate cluster of symptoms to these. Therefore, the content and organization of psychotic experiences in this clinical population is not indicative or predictive of illness severity or longevity, and supports the proposition that these experiences are part of the normal distribution of diverse thinking.

Sheffield and colleagues^12^ investigated the relationship between cognitive biases, delusional thinking, and game-based decision-making between individuals with schizophrenia spectrum conditions and healthy controls. Interestingly, across multiple self-report measures and various games, both groups showed similar relationships between beliefs and behavior: specifically, volatile decision-making (e.g., changing strategies multiple times) was positively correlated with paranoid thinking, and hasty decision-making was related to having unusual beliefs (e.g., mind reading, alien abduction), in both individuals with schizophrenia spectrum conditions and healthy controls. These findings further support the idea that the same cognitive mechanisms contribute to individual differences in both clinical and non-clinical populations.

## Psychiatric versus healthy experience

Despite potential common mechanisms for these experiences, and their prevalence across clinical and non-clinical populations, **it is important to distinguish psychiatric symptoms from healthy experience**. Perhaps the single most important difference between clinically-relevant and clinically-irrelevant experience is its impact on quality of life.

Environmental and social factors importantly contribute to this impact: conspiracy beliefs under a ‘sacred canopy’ (i.e., a social support buffer) can benefit an individual to a similar extent as being part of a religious or social organization; but these beliefs can exacerbate psychopathological characteristics if social support is lacking^5^. As another example, individuals who purposefully seek hallucinatory experiences are able to choose the environment, onset, and duration of the event; and can attribute the experience to an explicable source, such as Ganzflicker^3^. Controlling an unusual experience in this way can neutralize its potential negative effects. On the other hand, individuals who experience clinical hallucinations have little to no control over their experiences and cannot easily attribute them to a known source. The onset, duration, and environment of the experience are unpredictable, and can be embarrassing (e.g., at a social event) or even dangerous (e.g., while driving). All of these factors can lead to an extreme negative reaction to hallucinations and a debilitating impact on quality of life. So although much of the behavioral and cognitive bases of these divergent experiences are ubiquitous, this does not mean that these experiences should be treated the same for both clinical and non-clinical populations. The critical questions, then, concern how and why such experiences might develop into psychiatric illness.

In summary, hallucinations and delusions are part of a healthy range of diverse experiences found throughout the general population, but they can develop into more severe symptoms of psychiatric illness. It is important to investigate both common mechanisms (that contribute to our understanding of their cognitive and neural bases) and distinguishing factors (that separate clinically-relevant from clinically irrelevant symptoms) to comprehensively elucidate these often misunderstood experiences. This Special Collection provides a showcase of the cutting-edge research that encompasses these objectives.
